# Black Garlic Extract Modulates Endothelin Expression and Ovulatory Function in Monosodium Glutamate Treated Rats

**DOI:** 10.1002/fsn3.4766

**Published:** 2025-01-19

**Authors:** Arzu Gezer, Şükran Yediel Aras, Mustafa Ozkaraca, Nurcan Kilic Baygutalp, Gülhande Gundogdu, Ebru Karadag Sari, Gürsel Bedir, Hilal Üstündağ

**Affiliations:** ^1^ Vocational School of Health Services Atatürk University Erzurum Türkiye; ^2^ Graduate School of Natural and Applied Sciences, Pharmaceutical Research and Development Atatürk University Erzurum Türkiye; ^3^ Faculty of Health Sciences, Department of Midwifery Kafkas University Kars Türkiye; ^4^ Faculty of Veterinary Medicine, Department of Pathology Sivas Cumhuriyet University Sivas Türkiye; ^5^ Faculty of Pharmacy, Department of Biochemistry Atatürk University Erzurum Türkiye; ^6^ Faculty of Veterinary Medicine, Department of Histology and Embryology Kafkas University Kars Türkiye; ^7^ Faculty of Medicine, Department of Histology and Embryology Ataturk University Erzurum Türkiye; ^8^ Faculty of Medicine, Department of Physiology Erzincan Binali Yıldırım University Erzincan Türkiye

**Keywords:** black garlic, endothelin‐1, endothelin‐2, follicle, monosodium glutamate, ovary

## Abstract

Monosodium glutamate (MSG), a widely used food additive, has been associated with various health concerns, including potential reproductive toxicity. This study investigated the protective effects of black garlic (BG) ethanol extract against MSG‐induced ovarian damage in rats. Thirty‐two female rats in estrus were randomly divided into four groups (*n* = 8 per group): control (saline), BG (250 mg/kg BW), MSG (4 mg/g BW), and BG+MSG (combined treatment). Treatments were administered daily for 14 days. Ovarian tissues were collected for histopathological, immunohistochemical (IHC), and biochemical analyses. Histopathological examination revealed a significant reduction in cystic follicles in the BG+MSG group compared to the MSG group (*p* < 0.0001). IHC analysis showed decreased immunoreactivity of endothelin‐1 and endothelin‐2 in the BG+MSG group compared to the MSG group (both *p* < 0.01). Biochemical assays demonstrated significantly increased follicle‐stimulating hormone (FSH), luteinizing hormone (LH), and estradiol levels in the BG+MSG group compared to the MSG group (all *p* < 0.05), while progesterone levels were significantly lower in the MSG group compared to the BG+MSG group (*p* < 0.05). These findings suggest that BG ethanol extract may mitigate MSG‐induced ovarian dysfunction in rats by alleviating degenerative changes in follicles and modulating hormonal levels. This study provides insights into potential natural interventions for MSG‐related reproductive toxicity.

## Graphical abstract

Black garlic ethanol extract mitigated MSG‐induced ovarian dysfunction by reducing endothelin‐1 and endothelin‐2 expression, decreased cystic follicles and progesterone levels, while increased FSH, LH, and estradiol levels in rat ovarian tissue. These findings demonstrated BG extract's potential therapeutic role in protecting ovarian function against MSG toxicity.

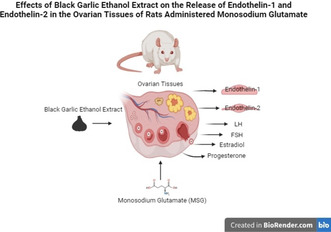

## 1 Introduction

Monosodium glutamate (MSG), known by the E 621 code, is a widely used food additive, particularly prevalent in Asian cuisines. Its increasing use in the food industry has raised concerns about potential health risks to consumers (Cooper et al. 2022). The American Federation of Experimental Biology has reported that MSG exposure at doses ranging from 0.5 to 2.4 g can lead to a condition known as “MSG symptom complex,” characterized by symptoms such as flushing, headache, and numbness (Adeleke et al. 2022).

Recent studies have suggested that excessive MSG consumption may be associated with various health issues. These include metabolic disorders such as increased body mass index, obesity, and insulin resistance (Shannon et al. 2017). Moreover, MSG has been linked to the exacerbation of neurological disorders, including Alzheimer's and Parkinson's diseases (Singh and Panda 2024). Particularly concerning are the reported harmful effects on the liver, reproductive, endocrine, and urinary systems (Tolulope et al. 2024). In female reproductive organs, MSG has been shown to negatively impact both structure and hormone levels (Abdulghani 2022).

In contrast, black garlic (BG), a processed fermented product, has gained attention for its potential health benefits. Produced by subjecting natural garlic bulbs to high temperatures (60°C and above) and relative humidity levels (85%–90%) for 30–40 days, BG results in odorless, sweet, and elastic black cloves (Javed 2022). Garlic extract has been reported to stimulate ovarian hormone secretion, activate the pituitary gland, and enhance estrogen receptor function (Jafari, Khalilzadeh, and Nejatbakhsh 2023).

The reproductive system's function is intricately regulated by various hormones and signaling molecules. Follicle‐stimulating hormone (FSH) and luteinizing hormone (LH) play crucial roles in folliculogenesis, oocyte selection, and sex steroid hormone synthesis. FSH stimulates ovarian follicle development in granulosa cells, while LH contributes to follicle development and maturation. Any deficiency or dysfunction in LH or FSH production or their effects can significantly impact female fertility (Casarini and Crépieux 2019).

Estrogens, primarily synthesized from cholesterol, support the development, maturation, and function of the female reproductive system and secondary sexual characteristics (Kinnear et al. 2020). Progesterone, synthesized mainly by the corpus luteum and placenta, prepares the endometrium for implantation and sustains pregnancy by inhibiting uterine contractions (Alqudah et al. 2022).

Endothelins, particularly endothelin‐1 and endothelin‐2, play significant roles in ovarian function. While structurally similar, they have distinct roles and distributions in the ovaries (Ko et al. 2022). Endothelin‐1 regulates steroidogenesis, vascular balance, and cell proliferation in the ovaries, although it inhibits progesterone production in luteal cells (Gupta 2022). Endothelin‐2, a 21‐amino acid peptide hormone, is crucial for ovulation. It is temporarily produced by granulosa cells of mature follicles and stimulates contractions in the myofibroblasts of the theca layer, facilitating follicle rupture. Additionally, endothelin‐2 can stimulate nitric oxide synthesis, leading to vasodilation and enhanced vascular permeability (Genovesi et al. 2022). Interestingly, estradiol treatment has been shown to increase endothelin‐2 expression (Hohos et al. 2021).

Despite the growing body of research on MSG's effects on the female reproductive system, there is a notable lack of comprehensive studies investigating the combined impact of MSG and BG. This study aims to explore the possible protective effects of BG in ovarian tissues against the deleterious effects of MSG.

While no studies with an identical protocol to the current study have reported on BG's protective effect against MSG's deleterious impacts on the reproductive system, a few experimental studies targeting the nervous system have indicated a protective effect of BG on MSG toxicity (Ahangar‐Sirous et al. 2022; Nurmasitoh, Sari, and Partadiredja 2018). This research gap underscores the novelty and potential significance of our study.

In light of the potential harmful effects of MSG on the female reproductive system and the promising health benefits of BG, this study aimed to investigate the possible protective effects of BG against MSG‐induced ovarian toxicity. Specifically, we sought to evaluate the impacts on ovarian histology, hormone levels (FSH, LH, estradiol, and progesterone), and the expression of endothelin‐1 and endothelin‐2 in rat ovarian tissue. By examining these parameters, we aimed to provide insights into the mechanisms by which BG might mitigate the deleterious effects of MSG on ovarian function. This research contributes to our understanding of ovulation regulation, potential treatments for MSG‐induced reproductive issues, and the development of novel approaches to support reproductive health. Furthermore, this study bridges a significant gap in the literature by exploring the combined effects of MSG and BG on the female reproductive system, offering valuable information for future research in reproductive endocrinology and toxicology.

## 2 Materials and Methods

### 2.1 BG Collection and Extraction

The BG from Edovital (Kastamonu, Türkiye) was dried, ground, and subjected to Soxhlet extraction. Ground garlic (50 g) was placed in an extraction cartridge with 96% ethanol (650 mL). The extraction lasted for 10 h, with continuous siphoning until the solvent was removed. The liquid extracts were filtered, evaporated, and stored at +4°C after precise weighing and desiccation for 12 h to remove residual solvent (Gündoğdu et al. 2023).

### 2.2 Animals and Experimental Model

The study received approval from the Animal Experiments Local Ethics Committee of Atatürk University, Faculty of Veterinary Medicine, under ethics committee number 11/27 on 12/28/2021, in line with the Declaration of Helsinki. Female *Sprague*–*Dawley* rats (*n* = 32, 8 per group), aged 3 months and weighing 250 ± 10 g, were obtained from Atatürk University Medical Experimental Research and Application Center. The rats were housed in standard cages with *ad libitum* access to tap water under a 12‐h light–dark cycle. Estrous cycles were monitored via vaginal smear, and only rats in the estrus phase and nulliparous rats were included. Four groups were formed:

#### 2.2.1 Control Group

Rats received a physiological dose of 1 mL saline orally every other day for a total of 14 days.

#### 2.2.2 BG Group

Rats received daily oral gavage of BG extract at 250 mg/kg body weight for 14 days (Amor et al. 2019).

#### 2.2.3 MSG Group

Rats received intraperitoneal injections of MSG at 4 mg/g body weight daily for 14 days (Haddad, Esmail, and Khazali 2021).

#### 2.2.4 BG+MSG Group

Rats received BG extract orally for 14 days followed by MSG (4 mg/g body weight daily for 14 days) injections 30 min later.

### 2.3 Tissue Collection

Rats were deeply sedated using a combination of 20 mg/kg thiopental sodium (IE Ulagay‐Türkiye) and 5% sevoflurane (Sevorane, Abbott Lab. Istanbul, Türkiye) inhalation anesthesia prior to blood sample collection. Following blood sampling, euthanasia was performed using a high dose of thiopental sodium (50 mg/kg). Both ovaries were carefully removed: the right ovary was immediately stored at −80°C for subsequent biochemical analyses, while the left ovary was fixed in 10% formaldehyde solution for histopathological examinations and embedded in paraffin.

### 2.4 Collection and Staining of Vaginal Smears

The vaginal smear technique was used for sample collection, followed by vaginal lavage using a plastic pipette containing 0.9% NaCl solution. A drop of the obtained suspension was placed on a slide daily, and after drying, the smears were stained with methylene blue. Analysis of the stained smear samples was conducted using an Olympus light microscope (×100 resolution; Olympus Scientific Solutions, Shinjuku, Tokyo, Japan) (Suci, Dalimunthe, and Syahputra 2023).

### 2.5 Histopathological Examination

The tissues were subjected to standard alcohol–xylene processing procedures (Isolab, Türkiye, CAS No: 64‐17‐5) and were then embedded in paraffin wax. Sections measuring 5 μm in thickness were placed onto polylysine‐coated slides and stained with hematoxylin and eosin (H&E) to evaluate cystic changes in ovarian follicles in randomly selected areas (Gezer, Laloglu, and Bölükbaş 2023; Gezer et al. 2024).

### 2.6 Immunohistochemical Examination

The tissue sections were subjected to xylene and alcohol washes followed with PBS rinsing. Endogenous peroxidase was neutralized with 3% H_2_O_2_ for 10 min. Antigen retrieval was performed by microwaving at 800 W for 10 min. The sections were then incubated overnight at 4°C with primary antibodies against endothelin‐1 (Abcam, Catalog No: ab2786) and endothelin‐2 (Origene, Catalog No: TA323082) at a 1:250 dilution. For secondary detection, an HRP system (Thermo Fisher, Catalog No: TP‐125‐HL) with DAB as the chromogen was used. Mayer's hematoxylin was used for counterstaining. Immunopositivity was semiquantitatively assessed as negative (−), weakly positive (+), moderate (++), or strongly positive (+++) (Gezer, Laloglu, and Bölükbaş 2023).

### 2.7 Biochemical Examination

Ovary samples were stored at −80°C until analysis. Tissue grinding was performed using liquid nitrogen in a TissueLyser (Qiagen, Hilden, Germany). Then, 50 mg of dry tissue was combined with 1 mL of PBS, followed by centrifugation. The tissue levels of FSH (BT Lab, EA0015Ra), LH (BT Lab, EA0013Ra), 17β‐estradiol (BT Lab, E1393Ra), and progesterone (BT Lab, EA0063Ra) were measured using ELISA kits according to the manufacturer's instructions.

### 2.8 Statistical Analysis

Histopathological and immunohistochemical (IHC) data were analyzed using IBM SPSS Statistics version 25.0 (IBM Corp., Armonk, NY, USA). Descriptive statistics were computed, and findings are reported as the mean ± SD and median (min–max). The normality of the data distribution was assessed via the Kolmogorov–Smirnov test. Group disparities were evaluated employing the nonparametric Kruskal–Wallis test, with specific group differences identified through the Mann–Whitney *U* test. Cyst counts were compared utilizing one‐way analysis of variance (ANOVA) (*p <* 0.05).

## 3 Results

### 3.1 Histopathological Results

According to the histopathological evaluation, statistically significant differences were detected among the groups (Table 1, *p <* 0.05).

**TABLE 1:** Comparisons of the percentage of cystic follicles in the control, BG, MSG, and BG+MSG groups.

	Cystic follicles (%)
Groups	
Control	0.16 ± 0.40^a^
BG	0.33 ± 0.51^a^
MSG	2.83 ± 0.40^b^
BG+MSG	1.83 ± 0.40^c^
*p*	< 0.01

*Note:* ^a,b,c^ Significant differences (*p <* 0.05) between groups (post hoc Tukey test results): ^a^ Significant difference between the control and the other groups, ^b^ Significant difference between the MSG and the other groups, ^c^ Significant difference between the BG +MSG and other groups.

Abbreviations: BG, black garlic (250 mg/kg body weight); BG+MSG, black garlic+monosodium glutamate; MSG, monosodium glutamate (4 mg/g body weight).

**FIGURE 1:**
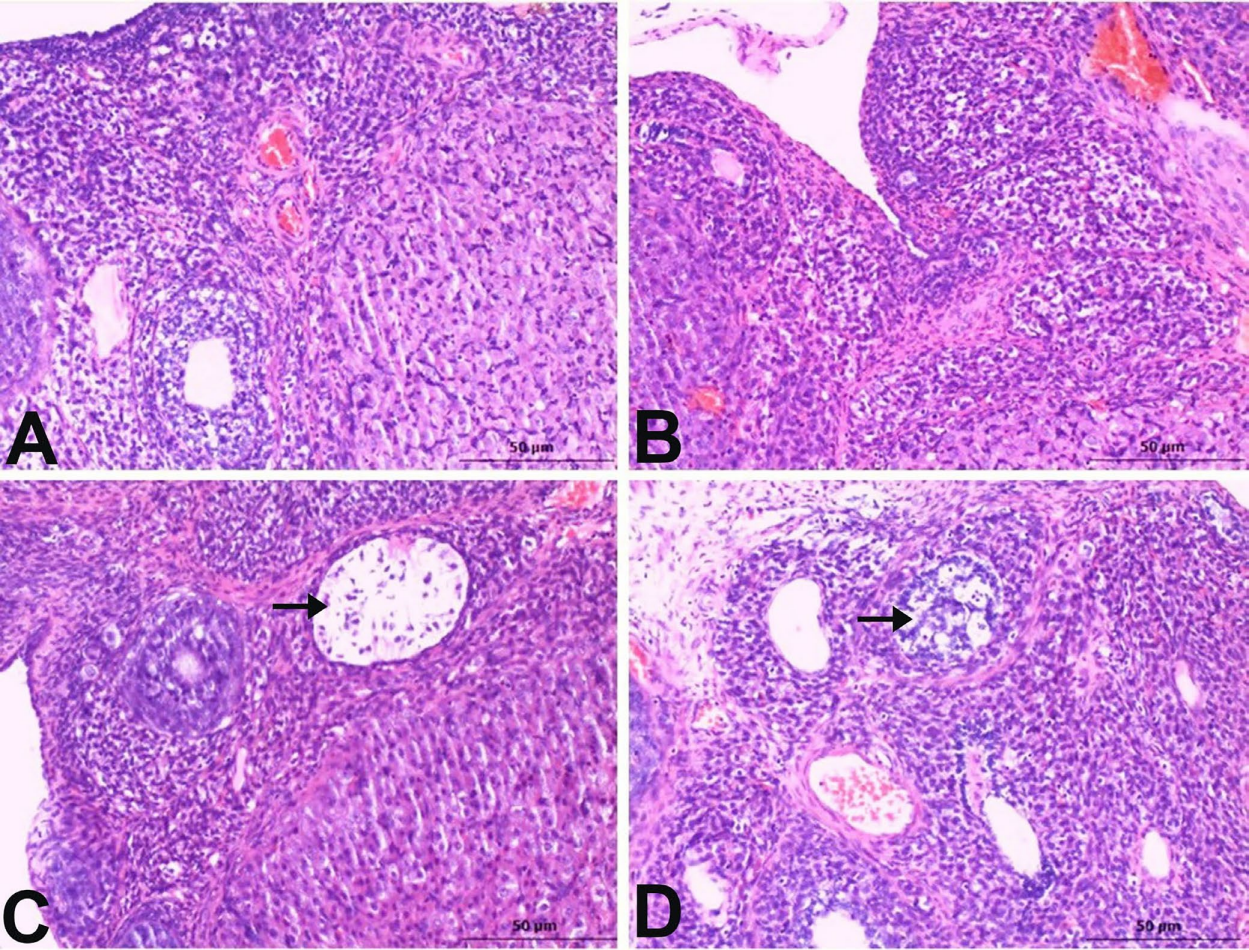
Histopathological examination of ovarian tissues in control, black garlic (BG), monosodium glutamate (MSG), and BG+MSG treated groups. (A) Control group showed normal ovarian histology. (B) BG group (250 mg/kg body weight) displayed normal ovarian structure similar to the control. (C) MSG group (4 mg/g body weight) exhibited severe cystic changes (arrow) with extensive cystic follicles of various sizes. (D) BG+MSG group showed moderate cystic changes (arrow) with a thinner granulosa cell layer in cystic follicles compared to the MSG group. Hematoxylin and eosin staining. Scale bar = 50 μm.

The ovarian tissues of the control and BG groups displayed a normal histological appearance (Figure 1A,B). Severe cystic changes were observed in the MSG group (Figure 1C), while moderate cystic changes were observed in the BG+MSG group. Moreover, the integrity of the cells was disrupted, and their normal arrangement was lost in the follicles with cystic changes (Figure 1D).

Compared with other follicles, cystic follicles have histologically different wall structures. These cystic follicles, particularly those found in the ovarian cortex, can be distinguished from other healthy follicles due to apoptotic and necrotic changes surrounding granulosa cells. The MSG group exhibited extensive cystic follicles of various sizes in the ovarian tissue, along with follicles at various developmental stages within the cortex. Graafian follicles were underdeveloped, and in both the MSG and BG+MSG groups, the granulosa cell layer was thin in the cystic follicles (Figure 1C,D).

A notable distinction was observed in the assessment of the number of cystic follicles among the groups (*p <* 0.0001). Moreover, there was no notable difference between the control and BG groups. Notable differences were observed among the control, MSG, and BG+MSG groups; the number of cystic follicles increased in the MSG group compared to the control and BG groups (both *p <* 0.0001). The number of cystic follicles in the BG+MSG group was lower than that in the MSG group (*p <* 0.0001), but it was greater than that in the control and BG groups (both *p <* 0.0001) (Figure 1C,D).

### 3.2 IHC Results

Significant differences were detected among the groups in terms of IHC staining for endothelin‐1 and endothelin‐2 (Table 2; *p <* 0.05).

**TABLE 2:** Comparisons of endothelin‐1 and endothelin‐2 immunopositivity in the secondary follicles and corpus luteum of rats in the control, BG, MSG, and BG+MSG groups.

	Endothelin‐1 immunopositivity (%) in secondary follicles	Endothelin‐2 immunopositivity (%) in secondary follicles	Endothelin‐1 immunopositivity (%) in corpus luteum	Endothelin‐2 immunopositivity (%) in corpus luteum
Groups				
Control	0.16 ± 0.40^a^	0.33 ± 0.52^a^	0.00 ± 0.00^a^	0.00 ± 0.00^a^
BG	0.16 ± 0.40^a^	0.33 ± 0.51^a^	0.00 ± 0.00^a^	0.00 ± 0.00 ^a^
MSG	2.83 ± 0.40^b^	2.83 ± 0.40^b^	1.00 ± 0.00^b^	1.83 ± 0.40^b^
BG+MSG	1.00 ± 0.00^c^	1.16 ± 0.40^c^	0.83 ± 0.40^b^	1.66 ± 0.5^b^
*p*	< 0.01	< 0.01	< 0.01	< 0.01

*Note:* ^a^Significant difference between the control and other groups, ^b^Significant difference between the MSG and the other groups, ^c^Significant difference between the BG and the other groups.

Abbreviations: BG, black garlic (250 mg/kg body weight); BG+MSG, black garlic+monosodium glutamate; MSG, monosodium glutamate (4 mg/g body weight); *p*, One‐way ANOVA test *p* value.

**FIGURE 2:**
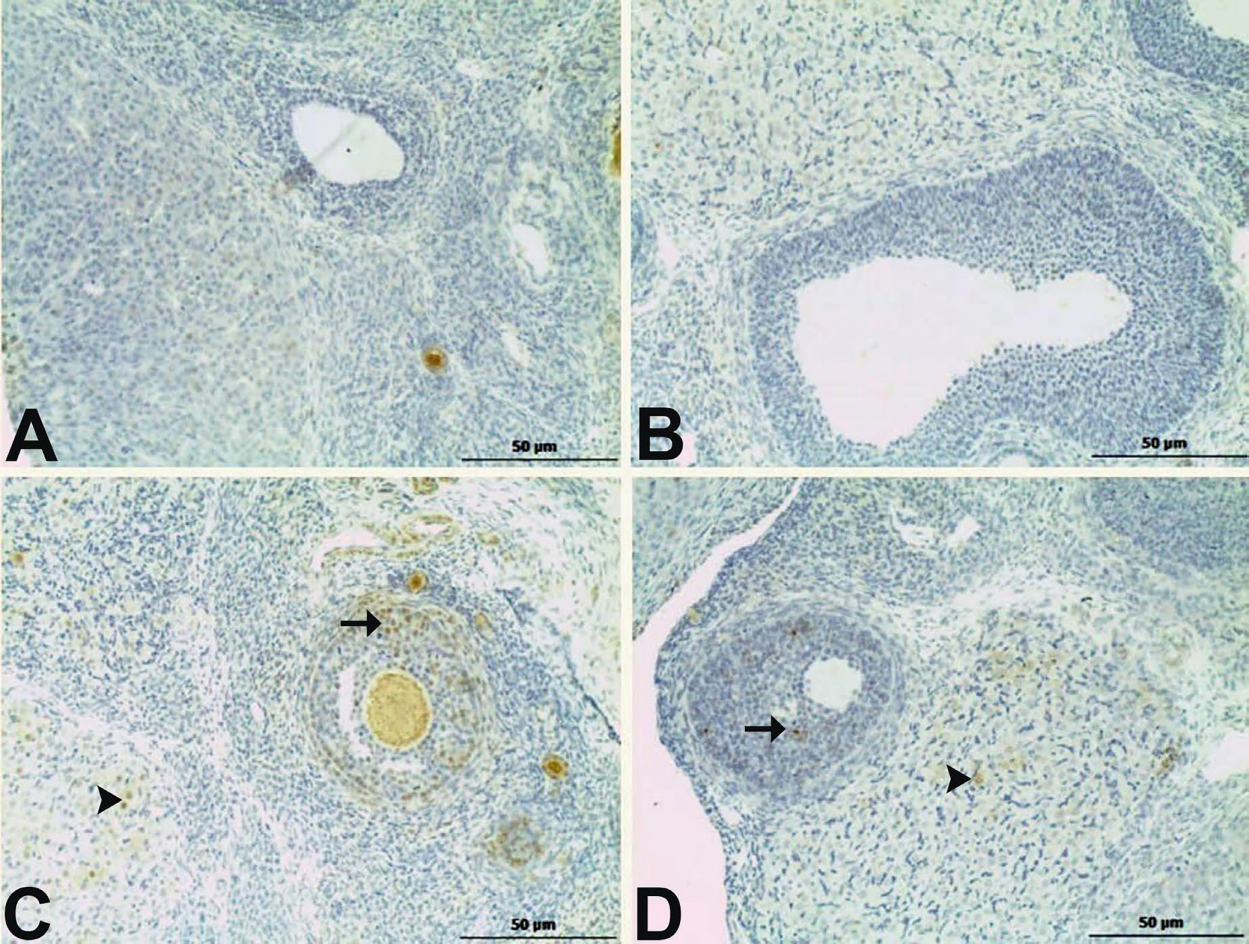
Immunohistochemical staining for endothelin‐1 in ovarian tissues of control, black garlic (BG), monosodium glutamate (MSG), and BG+MSG treated groups. (A) Control group and (B) BG group (250 mg/kg body weight) showed no significant positive reactions. (C) MSG group (4 mg/g body weight) displayed intense positive reactions in granulosa cells of secondary follicles (arrow) and mild positive reactions in corpus luteum (arrowhead). (D) BG+MSG group exhibited mild positive reactions in both granulosa cells of secondary follicles (arrow) and corpus luteum (arrowhead). Scale bar = 50 μm.

No significant positive reactions were observed for endothelin‐1 staining in the control or BG groups. In the MSG group, intense positive reactions were observed in the granulosa cells of the secondary follicles, while mild positive reactions were observed in the corpus luteum. In the BG+MSG group, mild positive reactions were detected in both the granulosa cells of the secondary follicles and the corpus luteum (Figure 2).

**FIGURE 3:**
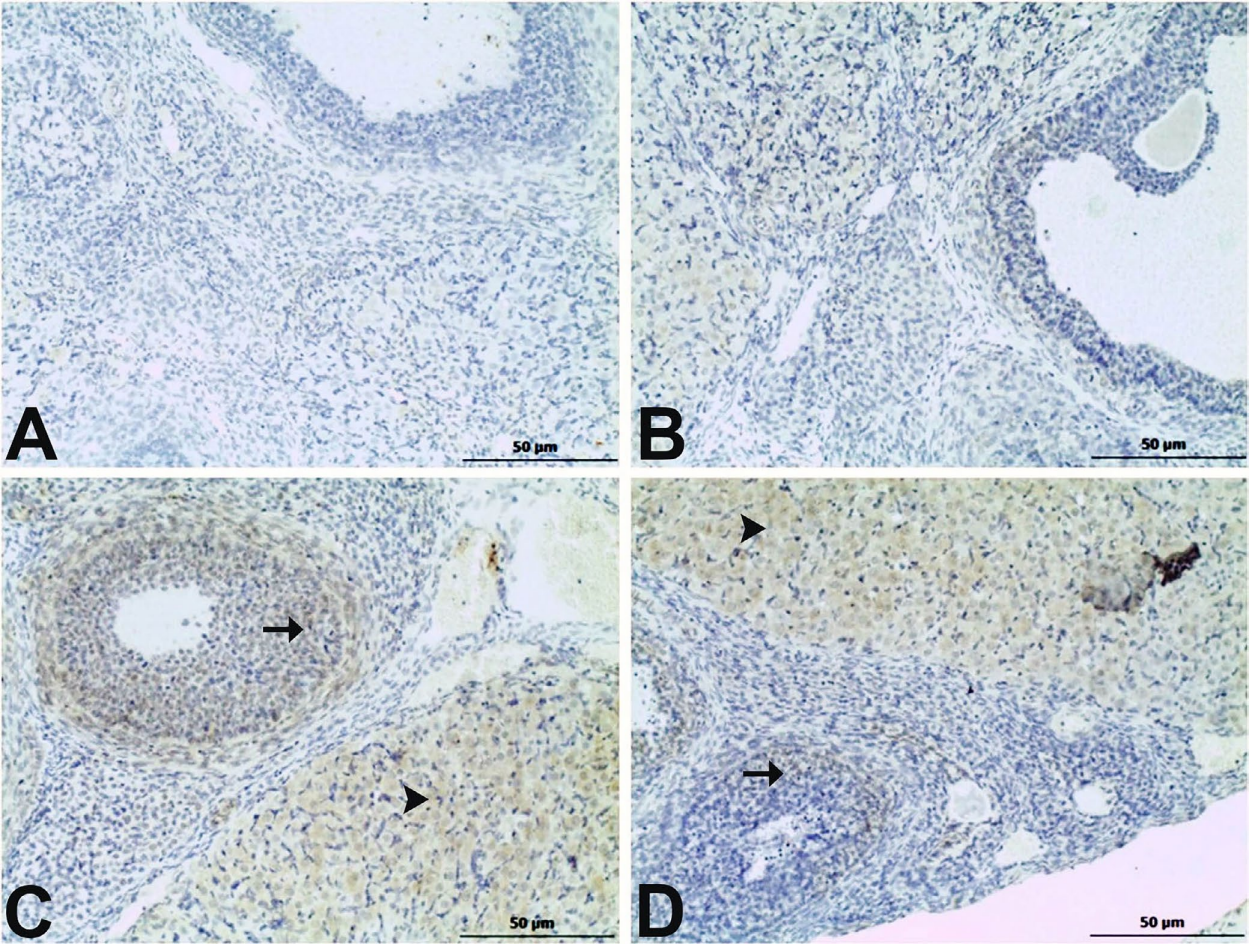
Immunohistochemical staining for endothelin‐2 in ovarian tissues of control, black garlic (BG), monosodium glutamate (MSG), and BG+MSG treated groups. (A) Control group and (B) BG group (250 mg/kg body weight) showed no significant positive reactions. (C) MSG group (4 mg/g body weight) displayed intense positive reactions in granulosa cells of secondary follicles (arrow) and moderate positive reactions in corpus luteum (arrowhead). (D) BG+MSG group exhibited mild positive reactions in granulosa cells of secondary follicles (arrow) and moderate positive reactions in corpus luteum (arrowhead). Scale bar = 50 μm.

Regarding endothelin‐2 staining, findings similar to those of endothelin‐1 were observed. No significant positive reactions were detected in the control or BG groups. In the MSG group, intense positive reactions were detected in the granulosa cells of the secondary follicles, while mild positive reactions were observed in the BG+MSG group. In both the MSG and BG+MSG groups, positive reactions in the corpus luteum were of moderate intensity (Figure 3).

### 3.3 Biochemical Results

**FIGURE 4:**
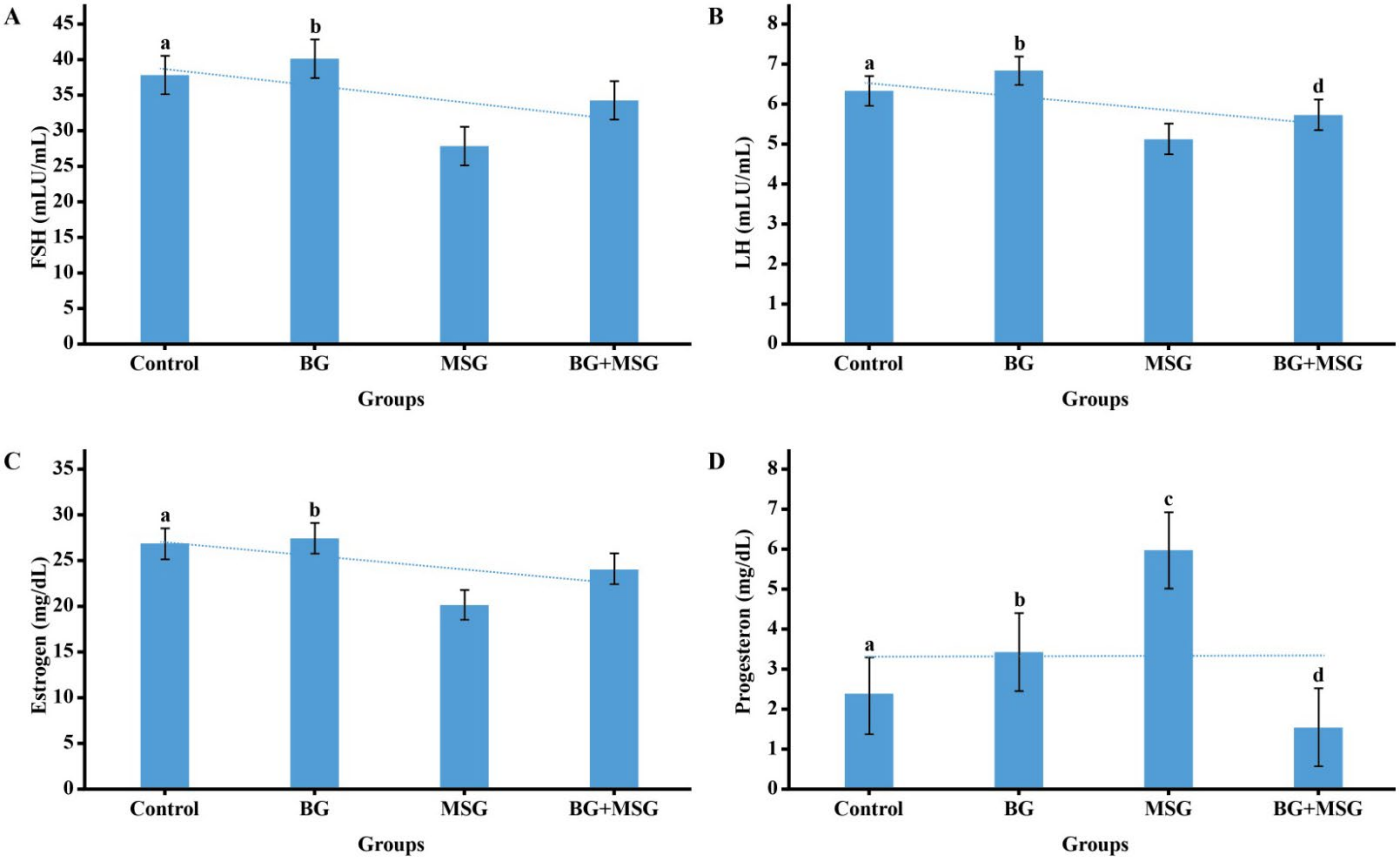
Tissue hormone levels in control, black garlic (BG), monosodium glutamate (MSG), and BG+MSG treated groups. (A) FSH levels, (B) LH levels, (C) Estradiol levels, and (D) Progesterone levels. Data are presented as mean ± SD. Different letters (a, b, c, d) indicate significant differences between groups (*p* < 0.05). BG, Black garlic (250 mg/kg body weight); BG+MSG, Black garlic + monosodium glutamate; MSG, Monosodium glutamate (4 mg/g body weight).

As shown in Figure 4A–D tissue hormone levels showed significant differences between groups for all measured parameters (*p* < 0.05).

FSH levels were significantly higher in the MSG group compared to both the control and BG groups. The BG+MSG group showed reduced FSH levels compared to the MSG group.

LH levels followed a similar pattern, with the MSG group showing the highest levels, significantly different from the control and BG groups (*p* < 0.05). The BG+MSG group demonstrated intermediate levels.

Estradiol levels were significantly elevated in the MSG group compared to the control and BG groups (*p* < 0.05), while the BG+MSG group showed partial normalization.

Progesterone levels were markedly decreased in the MSG group compared to both control and BG groups (*p* < 0.05). The BG+MSG group showed intermediate levels, suggesting partial protection by BG treatment.

Post hoc analyses revealed significant differences between the control and MSG groups, between BG and MSG groups for all hormones, and between MSG and BG+MSG groups for progesterone levels (*p* < 0.05 for all comparisons).

## 4 Discussion

In the present study, we investigated the protective effect of black garlic (BG) extract against monosodium glutamate (MSG)‐induced damage in rat ovarian tissue. Our key findings include decreased immunopositivity for endothelin‐1 and endothelin‐2, increased FSH, LH, and estradiol levels, and decreased progesterone levels following BG extract application. These results provide important insights into the potential protective role of BG against MSG‐induced ovarian toxicity and contribute to our understanding of the complex interplay between dietary compounds and reproductive health.

MSG inherently acts as an excitatory neurotransmitter in glutamatergic neurons in the brain, inhibiting reproductive function and increasing the activity of the hypothalamic–pituitary‐gonadal axis (Haddad, Esmail, and Khazali 2021). Our findings show that MSG administration is associated with alterations in FSH, LH, and estradiol levels along with changes in follicular morphology. While these changes share similarities with polycystic ovary patterns (Awad et al. 2023), it is important to note that hormonal increases alone cannot definitively indicate ovarian dysfunction (Casarini and Crépieux 2019). This is exemplified in clinical settings such as IVF treatment, where controlled ovarian stimulation deliberately increases gonadotropin levels to enhance follicular development (Fiorentino et al. 2023). MSG acts on glutamatergic neurons in the brain, affecting the hypothalamic‐pituitary‐gonadal axis through changes in LHRH and FSHRH production (Abdulghani 2022; Haddad, Esmail, and Khazali 2021). Similarly, BG influences hormone levels through activation of the pituitary gland and enhancement of estrogen receptor function (Jafari, Khalilzadeh, and Nejatbakhsh 2023). However, the overall impact on ovulatory function may differ between MSG and BG due to additional factors such as BG's antioxidant properties (Ahangar‐Sirous et al. 2022) and effects on the local tissue environment (Azordedast, Gholami‐Ahangaran, and Moghtadaei‐Khorasgani 2020). Previous studies have demonstrated the protective effects of antioxidants on reproductive tissue (Gezer et al. 2024; Pala et al. 2016), supporting the potential beneficial role of BG's antioxidant properties. Future studies incorporating specific markers of tissue function would be valuable to better understand these differential effects on ovarian function.

Our study revealed significant changes in endothelin‐1 and endothelin‐2 immunopositivity following BG treatment. These findings underscore the importance of the endothelin system in ovarian function and its potential role in mediating the protective effects of BG. The endothelin system plays crucial role in regulating various aspects of ovarian physiology, including follicular development, ovulation, and luteal function. Previous research has demonstrated that the lack of endothelin‐2 expression in mouse models results in significantly fewer oocytes and smaller offspring (Abdulkareem and Almohaidi 2023). Endothelin‐2 is particularly crucial in regulating the reproductive system, becoming active during follicle growth and development (Shrestha et al. 2018). In human ovaries, both endothelin‐1 and endothelin‐2 peptides have been detected in follicular fluid samples, with endothelin‐2 levels increasing proportionally with oocyte maturity (Abdulkareem and Almohaidi 2023). The concentration of endothelin‐2 has been shown to be greater in the fluid of oocytes capable of fertilization or division, indicating an association between endothelin‐2 levels, oocyte maturity, and fertilization potential (Fiorentino et al. 2023). This observation suggests that the modulation of endothelin levels by BG may contribute to its protective effects on ovarian function and fertility.

Our results demonstrated that BG may have protective effects against MSG‐induced ovarian damage. This finding is consistent with previous research showing that garlic can partially correct imbalances in estrogen metabolism and stimulate the release of gonadotropins and ovarian hormones in MSG‐induced fibroids (Falahatian, Haddad, and Pakravan 2022). The protective effects of BG observed in our study may be attributed to its rich content of bioactive compounds, including organosulfur compounds, flavonoids, and antioxidants.

In our study, higher numbers of corpora lutea were observed in the control and BG groups compared to other groups. Together with the observed higher progesterone levels in these groups, these findings suggest differences in ovarian function between treatment groups. The MSG group showed reduced numbers of corpora lutea and lower progesterone levels, while in the BG+MSG group, both parameters showed improvement compared to the MSG group. These observations, while not definitive evidence of ovulation, suggest that BG treatment may have a protective effect on ovarian function. The combination of histological observations and hormone measurements provides insights into how BG might help maintain normal ovarian function against MSG‐induced changes. However, further studies incorporating pregnancy outcomes would be needed to definitively assess the impact on ovulation.

Our results of increased FSH, LH, and estradiol levels, along with decreased progesterone levels following BG treatment, are supported by previous studies. Garlic supplementation has been shown to increase the release of gonadotropins and ovarian hormones through activation of the pituitary gland and augmentation of estrogen receptor release (Azordedast, Gholami‐Ahangaran, and Moghtadaei‐Khorasgani 2020). Additionally, garlic extract has been shown to increase LH and FSH levels in female mice exposed to heat stress (Jamadi et al. 2023).

These hormonal changes induced by BG may play a crucial role in counteracting the detrimental effects of MSG on ovarian function. The increased levels of FSH and LH may promote follicular development and ovulation, while the elevated estradiol levels may support overall reproductive health. The decreased progesterone levels observed in our study warrant further investigation to understand their significance in the context of BG's protective effects.

While our study provides valuable insights into the protective effects of BG against MSG‐induced ovarian damage, it is important to acknowledge its limitations. The focus on rat models, while providing important preliminary data, may not fully translate to human physiology. Therefore, future research should aim to validate these findings in human studies or more advanced in vitro models of human ovarian tissue. Additionally, long‐term studies are needed to assess the safety and efficacy of BG supplementation in protecting against MSG‐induced ovarian damage over extended periods. Such studies could provide valuable information on the optimal dosage and duration of BG supplementation for maximum protective effects.

## 5 Conclusion

This study investigated the protective effects of BG extract against MSG‐induced ovarian damage in rats. Our findings demonstrated that BG application decreased immunopositivity for endothelin‐1 and endothelin‐2, increased FSH, LH, and estradiol levels, while decreasing progesterone levels. Furthermore, we observed an increase in the number of corpora lutea and a reduction in cystic follicles in the BG+MSG group compared to the MSG‐only group. These results suggest that BG may exert protective effects against MSG‐induced ovarian damage, potentially through modulation of the endothelin system and regulation of reproductive hormones. While these findings are promising, further research is needed to elucidate the precise molecular mechanisms underlying BG's protective effects and validate these results in human studies. This research contributes to our understanding of the potential use of natural compounds in mitigating reproductive toxicity and may inform future strategies for protecting ovarian function against environmental toxins.

## Author Contributions

**Arzu Gezer:** conceptualization (equal), funding acquisition (equal), investigation (equal), methodology (equal), resources (equal), writing – original draft (equal), writing – review and editing (equal). **Şükran Yediel Aras:** conceptualization (equal), investigation (equal), methodology (equal), writing – original draft (equal), writing – review and editing (equal). **Mustafa Ozkaraca:** formal analysis (equal), methodology (equal), writing – original draft (equal), writing – review and editing (equal). **Nurcan Kilic Baygutalp:** conceptualization (equal), formal analysis (equal), methodology (equal), resources (equal), software (equal), writing – original draft (equal), writing – review and editing (equal). **Gülhande Gundogdu:** investigation (equal), methodology (equal), resources (equal), writing – original draft (equal). **Ebru Karadag Sari:** conceptualization (equal), supervision (equal), writing – original draft (equal), writing – review and editing (equal). **Gürsel Bedir:** conceptualization (equal), data curation (equal), investigation (equal), methodology (equal), writing – original draft (equal). **Hilal Üstündağ:** conceptualization (equal), methodology (equal), supervision (equal), writing – original draft (equal), writing – review and editing (equal).

## Conflicts of Interest

The authors declare no conflicts of interest.

## Data Availability

The data that support the findings of this study are available from the corresponding author upon reasonable request.
